# FMCerNet: frequency-aware multi-label learning for fine-grained cervical cytology screening

**DOI:** 10.1093/bioinformatics/btag759

**Published:** 2026-10-19

**Authors:** Linyun Zhou, Qiuyu Cai, Xiaoling Wang, Weihan Li, Jian Yang, Mingli Song, Jing Zhang, Feng Zhou, Qihan You, Yudong Wang, Zunlei Feng, Xiuming Zhang

**Affiliations:** College of Computer Science, Zhejiang University, Hangzhou, Zhejiang 310058, China; Department of Pathology, The First Affiliated Hospital, Zhejiang University School of Medicine, Hangzhou, Zhejiang 310003, China; Department of Pathology, The First Affiliated Hospital, Zhejiang University School of Medicine, Hangzhou, Zhejiang 310003, China; School of Software Technology, Zhejiang University, Ningbo, Zhejiang 315100, China; School of Software Technology, Zhejiang University, Ningbo, Zhejiang 315100, China; College of Computer Science, Zhejiang University, Hangzhou, Zhejiang 310058, China; Department of Pathology, The First Affiliated Hospital, Zhejiang University School of Medicine, Hangzhou, Zhejiang 310003, China; International Peace Maternal and Child Health Hospital, School of Medicine, Shanghai Jiao Tong University, Shanghai 200030, China; Department of Pathology, The First Affiliated Hospital, Zhejiang University School of Medicine, Hangzhou, Zhejiang 310003, China; International Peace Maternal and Child Health Hospital, School of Medicine, Shanghai Jiao Tong University, Shanghai 200030, China; School of Software Technology, Zhejiang University, Ningbo, Zhejiang 315100, China; Department of Pathology, The First Affiliated Hospital, Zhejiang University School of Medicine, Hangzhou, Zhejiang 310003, China

## Abstract

**Motivation:**

Automated cytology screening is essential for early cervical cancer prevention, where gigapixel whole-slide images (WSIs) are typically partitioned into tiles for tile-level feature extraction and subsequent WSI-level diagnosis aggregation. However, existing tile-level methods remain constrained by limited input sizes, which frequently obscures sparse and morphologically subtle abnormal cells. Furthermore, these methods commonly formulate tile-level representation learning as a coarse single-label classification task, thereby failing to capture the complex co-occurrence patterns and morphological heterogeneity of cervical cytological abnormalities.

**Results:**

We propose the Frequency-Aware Multi-Label Learning Network (FMCerNet), a tile-level feature extractor equipped with a Large-Field Frequency-Enhanced ViT backbone and a Coarse-to-Fine multi-label classification head. The former incorporates a Dual-Tree Complex Wavelet Transform module to efficiently extract frequency-domain features from large-scale tiles, whereas the latter enhances fine-grained tile representation decoding through hierarchical supervision. Experiments on both public and private datasets demonstrate that FMCerNet improves the accuracy of positive screening and fine-grained subtype classification while maintaining high inference speed.

**Availability and implementation:**

Code at https://github.com/imAzhou/FMCerNet and archived on Zenodo (doi: 10.5281/zenodo.22886105).

## 1 Introduction

Cervical cancer is a leading cause of morbidity and mortality among women, ranking as the fourth most common cancer globally (Sung *et al.* 2021). Cervical cytology remains a primary screening modality for cervical cancer prevention and early detection, yet manual interpretation by cytopathologists is labor-intensive, subjective, and susceptible to inter-observer variability (Cohen *et al.* 2019, Wang *et al.* 2024a). With recent advances in deep learning (DL), increasing attention has focused on DL-based methods for whole-slide image (WSI) analysis (Murphy 2014, Marée *et al.* 2016, Jiang *et al.* 2023, Wang *et al.* 2024a). A typical pipeline follows an “extract-then-aggregate” paradigm, in which a feature extractor encodes tile regions into representations that are subsequently aggregated to generate the final WSI-level diagnosis (Ilse *et al.* 2018, Li *et al.* 2021, Shao *et al.* 2021, Tang *et al.* 2024, Mao *et al.* 2025). Within this pipeline, the discriminative power of the feature extractor is pivotal. Unlike cell-level feature extractors (Chen *et al.* 2022, Wang *et al.* 2024a, Jiang *et al.* 2025) that require exhaustive fine-grained annotations of cellular clusters, tile-level feature extractors operating on partitioned tiles offer a favorable trade-off between annotation cost and inference efficiency, making them highly suitable for practical clinical deployment. Recent tile-level works have shown promise: Cheng *et al.* (2021) utilized a multi-magnification strategy to identify suspicious regions. Zhao *et al.* (2022) demonstrated that foundation models with limited fine-tuning can achieve effective multi-class classification. And Stegmüller *et al.* (2024) combined unsupervised pre-training with tile-level supervision for robust performance. However, these methods exhibit intrinsic limitations in cervical cytology screening. First, their transformer-based backbones incur high computational cost, restricting input to small-scale tiles and limiting large-field morphological context while potentially sacrificing high-frequency cellular details, such as chromatin texture and nuclear membrane irregularities, that are critical for subtle lesion subtype differentiation. Moreover, tile-level training typically relies on simplified supervision, either binary labels (Stegmüller *et al.* 2024) or single-category labels assigned according to the most severe lesion (Zhao *et al.* 2022), thereby forcing the model to ignore the co-occurrence of heterogeneous lesion categories and learn feature representations with limited fine-grained semantic details.

These limitations suggest that WSI-level classification can be improved by a tile-level extractor that satisfies two key objectives: efficiently extracting high-frequency cytomorphological details from large-field tiles without imposing excessive computational overhead, and formulating representation learning as multi-label classification to capture non-mutually exclusive lesion semantics from co-occurring abnormal categories. For the first objective, frequency-domain transformations have been investigated as complements to vision transformer (ViT) (Dosovitskiy *et al.* 2021) to enrich spatial-domain representations with spectral information. Fourier-based approaches enable efficient global context mixing(Zeng *et al.* 2024a,b), while wavelet-based approaches provide multi-scale texture analysis (Yao *et al.* 2022, Meng *et al.* 2025). However, Fourier transforms have limited spatial localization for fine-grained texture characterization, whereas wavelet transforms suffer from shift variance and limited directional selectivity. These limitations make them suboptimal for cervical cytology screening. For the second objective, multi-label classification (MLC) has been extensively explored in natural image recognition (Zhu *et al.* 2017, Liu *et al.* 2021, Ridnik *et al.* 2023) and has also been introduced into several medical imaging tasks (Wang and Wei 2022, Tomko *et al.* 2023, Xie *et al.* 2024, Manalo *et al.* 2025). Nevertheless, MLC remains underexplored in cervical cytology screening, where fine-grained discrimination among visually similar lesion subtypes, such as LSIL and HSIL, requires subtle cytomorphological differences that standard global pooling or simple attention mechanisms often fail to preserve.

In this paper, we propose the Frequency-Aware Multi-Label learning network (FMCerNet), a tile-level feature extractor for fine-grained cervical cytology screening. FMCerNet is equipped with a Large-Field Frequency-Enhanced ViT (LFreq-ViT) backbone to efficiently process large-field inputs with low computational cost, and a Coarse-to-Fine (C2F) head that leverages salient maps from a coarse binary branch to guide fine-grained MLC. Specifically, as illustrated in Fig. 1b, the LFreq-ViT backbone processes each input tile at two resized scales, i.e. $224^{2}$ and $1024^{2}$. The small-scale input is fed into a ViT to extract high-level semantic context, whereas the large-scale input is processed by a frequency-aware module based on the Dual-Tree Complex Wavelet Transform (DTCWT). The latter first embeds the large-scale input into a 2D feature space and then uses the DTC wavelet to extract six orientation-selective high-frequency components, which are further refined to preserve details essential for differentiating lesion subtypes. The resulting semantic tokens and frequency-enhanced tokens are concatenated and fused through a linear projection before being passed to the C2F head. The C2F head provides hierarchical supervision through two complementary branches: (i) a binary branch based on CHIEF (Wang *et al.* 2024b) serves as a coarse binary classification to ensure high sensitivity to abnormal lesions, and (ii) an MLC branch equipped with the proposed Bilateral Attention Query (BiQuery) module to mine category specific semantics. Meanwhile, the token salient map generated by CHIEF guides feature decoding in MLC branch by reweighting and residually enhancing tile tokens before the tokens are queried by BiQuery. Joint supervision from the two branches produces discriminative tile representations, thereby enhancing downstream WSI-level prediction for both binary and multi-class classification. The main contributions are summarized:

**Figure 1:**
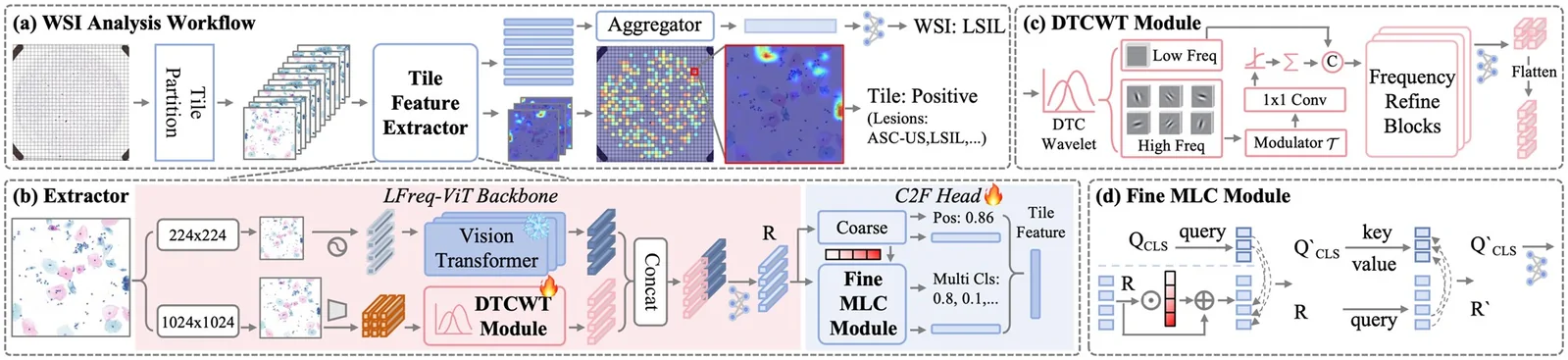
(a) Workflow for whole-slide image (WSI) analysis. (b) Structure of the proposed feature extractor, FMCerNet. (c) Frequency-aware feature extraction using the Dual-Tree Complex Wavelet Transform (DTCWT). (d) Architecture of the fine-grained multi-label classification module.

- FMCerNet introduces a multi-label tile-level learning paradigm that captures intra-tile cellular heterogeneity and co-occurring lesion categories for improved WSI-level analysis.
- A novel LFreq-ViT backbone is introduced, augmenting vision transformer models with frequency-domain transforms to support large-scale inputs and capture fine-grained details.
- A C2F head is designed to provide hierarchical supervision by jointly optimizing coarse binary and fine-grained MLC, leveraging coarse attention to guide feature decoding in the MLC branch.
- Extensive experiments show that FMCerNet notably improves tile-level and WSI-level performance, outperforming state-of-the-art methods in both positive screening and multi-class classification.

## 2 Materials and methods

This section presents the proposed tile-level feature extractor FMCerNet, consisting of a Large-Field Frequency-Enhanced ViT (LFreq-ViT) backbone and a Coarse-to-Fine (C2F) task head.

### 2.1 LFreq-ViT backbone

The quadratic complexity of self-attention with token length restricts most ViTs to limited input sizes (e.g. 224×224). To reduce the computational burden of gigapixel WSI analysis, large tiles (e.g. 800×800) are typically cropped from WSIs and resized to the model input size. However, such aggressive downsampling is particularly detrimental to cervical cytology screening, where sparse abnormal cells and subtle cytomorphological details are critical for differentiating lesion subtypes. For example, normal intermediate nuclei measure 5–10 µm, whereas ASC-US and LSIL nuclei are roughly 1.5–3 times larger. After about 4× downsampling, these size differences shrink to only a few pixels or even fade out, making fine-grained classification difficult. Here, LFreq-ViT is introduced to combine a ViT branch with a frequency transform branch, enabling efficient processing of large-field tile inputs with low computational overhead while preserving discriminative cytomorphological details.

#### 2.1.1 Two-scale workflow

As illustrated in Fig. 2a, the cropped tile is resized to a small-scale $I_{\mathrm{small}}\in R^{3\times 224\times 224}$ and then patch-embedded into a token embedding $E\in R^{N\times d}$, where *N* is the number of tokens and each token is a *d*-dimensional vector. The patch embedding layer and subsequent Transformer layers are initialized from a cytopathology foundation model pretrained through self-supervised learning on millions of unlabeled cytology tiles. To preserve the rich high-level contextual knowledge acquired during pretraining, all parameters of the ViT branch remain frozen throughout training. The resulting token embeddings are denoted as $\hat{E}\in R^{N\times d}$.

**Figure 2:**
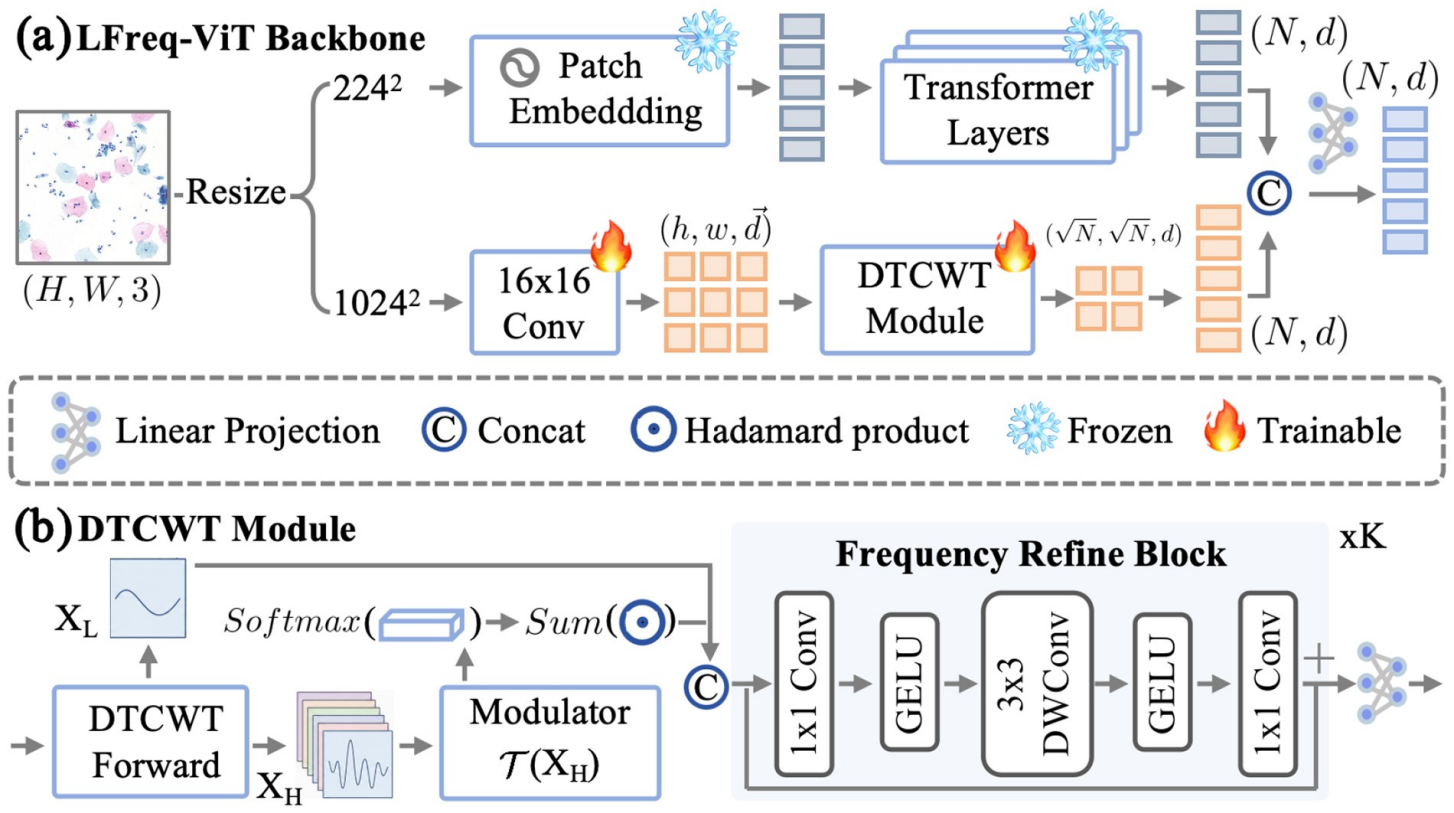
(a) The proposed two-scale LFreq-ViT backbone, where the upper branch processes small-scale inputs and the lower branch handles large-scale inputs. (b) The detailed structure of the proposed DTCWT module, where $X_{L}$ and $X_{H}$ denote the low- and high-frequency components, respectively.

In parallel, the cropped tile is resized to a large-scale input $I_{\mathrm{large}}\in R^{3\times 1024\times 1024}$ and projected into a feature map $X\in R^{h\times w\times \vec{d}}$ through a convolution layer, where *h* and *w* denote the spatial dimensions and $\vec{d}$ is the channel size. The DTCWT module $F_{\mathrm{dtcwt}}$ transforms *X* into frequency-enhanced token representations $\hat{X}\in R^{N\times d}$. Finally, the outputs of the small- and large-scale workflow are concatenated and passed through a linear projection layer to obtain the tile representation tokens $R\in R^{N\times d}$, which integrate high-level contextual semantics with fine-grained details, i.e. $R=\mathrm{Linear}(\text{Concat}(\hat{E},\hat{X}))$.

#### 2.1.2 DTCWT block

The Dual-Tree Complex Wavelet Transform (DTCWT) is a shift-invariant wavelet transform with limited redundancy, decomposing the input into six subbands with quadrature real and imaginary components, each approximately aligned with orientations of 15°, 45°, 75°, 105°, 135°, and 165° (Selesnick *et al.* 2005). By capturing low-level, fine-grained features across multiple orientations, the DTCWT provides a strong complementary representation to semantic features (Patro and Agneeswaran 2023), making it well suited for processing large-scale inputs in our backbone.

As shown in Fig. 2b, for a given input feature map $X\in R^{h\times w\times \vec{d}}$, the DTCWT forward transform decomposes the input into a low-frequency component $X_{L}\in R^{h/2\times w/2\times \vec{d}}$ and complex-valued high-frequency coefficients $X_{H}$ at six orientations. It is worth noting that the DTCWT module is implemented using a two-level wavelet decomposition. Consequently, the high-frequency coefficients consist of two groups of subbands at different spatial scales, denoted as $X_{H}^{1}\in R^{6\times h/2\times w/2\times \vec{d}\times 2}$ and $X_{H}^{2}\in R^{6\times h/4\times w/4\times \vec{d}\times 2}$, where 6 denotes the directional subbands, and the last dimension corresponds to the real $X_{\left(H,r\right)}^{l}$ and imaginary $X_{\left(H,i\right)}^{l}$ components, with l∈{1,2}. The high-frequency coefficients $X_{H}^{l}$ are then independently fed into a Modulator T to obtain stable and dynamically balanced directional representations $X_{H}^{l^{\prime }}\in R^{6\times h^{\prime }\times w^{\prime }\times \vec{d}}$ with $h^{\prime }=h/2^{l},w^{\prime }=w/2^{l}$:


(1) $$X_{H}^{l^{\prime }}=T(X_{\left(H,r\right)}^{l},X_{\left(H,i\right)}^{l})$$

(2) $$=\text{log}\;\left(1+\sqrt{{\left(X_{\left(H,r\right)}^{l}\right)}^{2}+{\left(X_{\left(H,i\right)}^{l}\right)}^{2}+\epsilon }\right),$$

where ϵ is a small constant for numerical stability. The magnitude operation produces phase-invariant responses, while the logarithmic transform stabilizes high-frequency representations through dynamic range compression. An orientation-aware aggregator is then applied to $X_{H}^{l^{\prime }}$ to adaptively emphasize informative directional responses while suppressing less relevant ones, producing a compact high-frequency representation ${\tilde{X}}_{H}^{l^{\prime }}\in R^{h^{\prime }\times w^{\prime }\times \vec{d}}$:


(3) $${\tilde{X}}_{H}^{l^{\prime }}=\sum_{t=1}^{6}(X_{H_{t}}^{l^{\prime }}⊙\mathrm{softmax}(\phi (X_{H_{t}}^{l^{\prime }}))),$$

where ⊙ denotes the Hadamard product, ϕ(·) denotes a 1×1 convolution, softmax(·) normalizes attention weights over the six orientations indexed by *t*. The low-frequency component $X_{L}$ and the modulated high-frequency representation ${\tilde{X}}_{H}^{l^{\prime }}$ are first aggregated as


(4) $$\bar{X}=\text{Concat}(X_{L},{\tilde{X}}_{H}^{1^{\prime }},\mathrm{Up}({\tilde{X}}_{H}^{2^{\prime }})),\;\bar{X}\in R^{h/2\times w/2\times 3\cdot \vec{d}},$$

Up(·) denotes bilinear upsampling. The representation $\bar{X}$ is refined by a stack of Frequency Refine Blocks, as illustrated in Fig. 2b. Due to feature concatenation, the first block operates on $3\cdot \vec{d}$ channels, while all others use $\vec{d}$ channels. After *K* refinement stages, the features undergo 2×2 average pooling with a stride of 2, followed by projection to dimension *d* and spatial flattening to obtain $\hat{X}\in R^{N\times d}$.

### 2.2 C2F head

For cervical cytology screening, the clinical objective is 2-fold: achieving high sensitivity and accurate subtype classification. Yet single-label tile-level learning collapses heterogeneous lesions into a single category, limiting the modeling of intra-class variation and co-occurring abnormalities. Although MLC naturally accommodates lesion co-occurrence, its complex output space often hinders the detection of diagnostically salient regions. The remarkable ability of CHIEF (Wang *et al.* 2024b) to localize diagnostically relevant regions motivates the design of our Coarse-to-Fine (C2F) head with a hierarchical supervision strategy. Specifically, CHIEF serves as a binary branch that acts as a coarse positive tile detector and generates attention maps to guide feature learning in a BiQuery-based MLC branch, which captures fine-grained category-specific semantics. Together, the two branches enable reliable binary screening and multi-label diagnosis while producing discriminative tile representations for downstream WSI-level prediction. The loss settings for joint training are detailed in Section A.3, available as supplementary data at *Bioinformatics* online.

#### 2.2.1 Coarse binary branch

As shown in Fig. 3a, the representation tokens $R\in R^{N\times d}$ are fed into the binary branch. The input *R* is first projected via $F_{\mathrm{proj}}$ into a lower-dimensional space, yielding $\tilde{R}\in R^{N\times D}$ with D=d/2. A gating mechanism is then applied to adaptively modulate $\tilde{R}$, producing an intermediate modulated representation $\tilde{A}$:


(5) $$\tilde{A}=\text{tanh}(G_{1}(\tilde{R}))⊙\sigma (G_{2}(\tilde{R})),\;\tilde{A}\in R^{N\times D},$$

where tanh(·) extracts discriminative features, σ(·) denotes sigmoid gating that suppresses irrelevant background, $G_{1}$ and $G_{2}$ denote two distinct learnable linear projections, G(x)=Wx+b with trainable parameters *W* and *b*. Then, another linear projection is applied to aggregate $\tilde{A}$ into a score vector $A=G(\tilde{A})$. The $A\in R^{N}$ plays a central role in three aspects: (i) aggregating representations into a vector V [Equation (6)], (ii) generating a coarse diagnostically salient map M [Equation (7)], and (iii) predicting the positive probability $\hat{p}$ [Equation (8)]:


(6) $$V=\mathrm{softmax}(A)\cdot \tilde{R},\;V\in R^{D},$$

(7) $$M=\sigma (A)⊙\sigma (G(\tilde{R})),\;M\in R^{N},$$

(8) $$\hat{p}=\sigma (G(V)).$$

**Figure 3:**
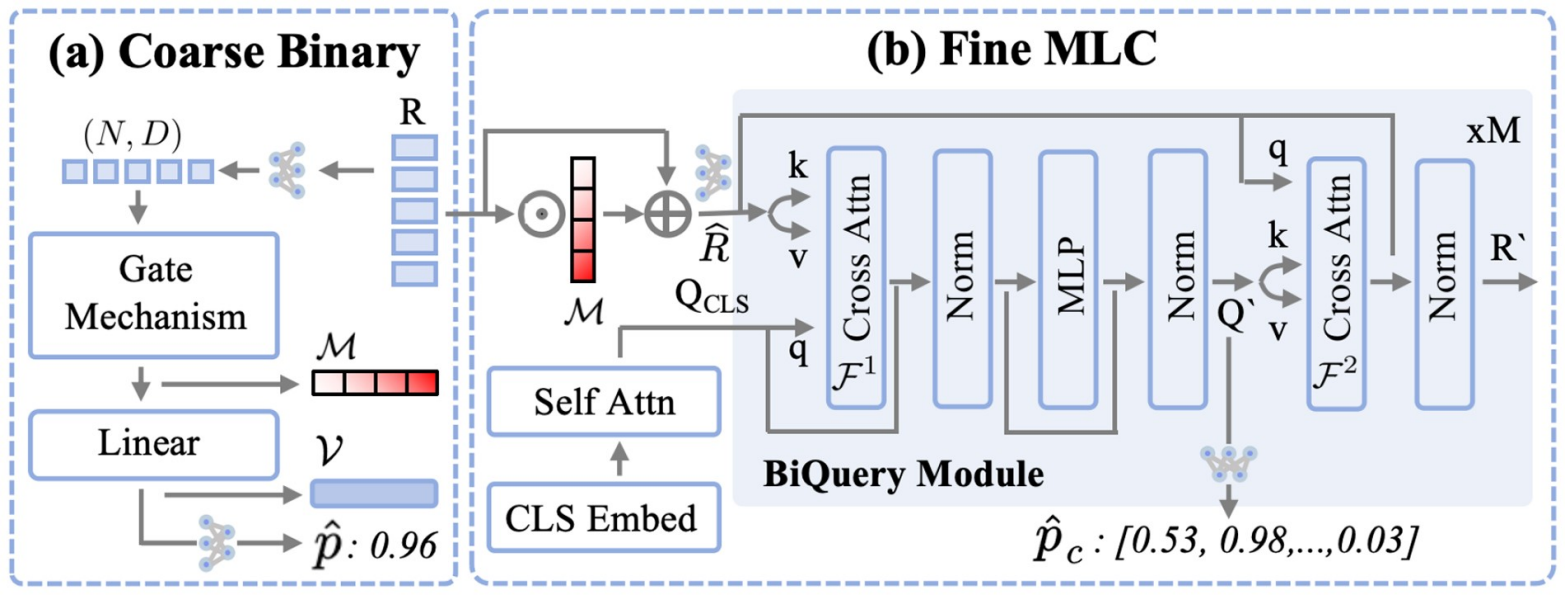
Workflow of the proposed dual-branch task head, consisting of (a) a binary branch and (b) a multi-label classification (MLC) branch.

The V forms part of the tile representation, while M weights features in the fine MLC branch to emphasize diagnostically salient regions.

#### 2.2.2 Fine MLC branch

On the other hand, the representation *R* is fed into the MLC branch for fine-grained classification, as shown in Fig. 3b. The map M generated by the coarse branch is used to reweight *R*, followed by a linear projection to obtain the representation $\hat{R}=G(R+R⊙M)$, where $\hat{R}\in R^{N\times D}$. Meanwhile, class embeddings $Q_{\mathrm{CLS}}\in R^{C\times D}$ are introduced as class query tokens, where *C* denotes the number of lesion categories. Inspired by SAM (Kirillov *et al.* 2023), we propose a Bilateral Attention Query (BiQuery) module which establishes bidirectional interactions between class and image tokens to enhance the learning of $Q_{\mathrm{CLS}}$. Specifically, the class queries $Q_{\mathrm{CLS}}$ first pass through a self-attention layer and are then fed into the BiQuery module together with representation $\hat{R}$. BiQuery consists of two groups of cross-attention modules, $F_{\mathrm{attn}}^{1}$ and $F_{\mathrm{attn}}^{2}$, producing the updated representations $Q_{\mathrm{CLS}}^{{\;}^{\prime }}$ and $R^{{\;}^{\prime }}$:


(9) $$Q_{\mathrm{CLS}}^{\prime }=F_{\mathrm{attn}}^{1}(Q_{\mathrm{CLS}},R),\;R^{\prime }=F_{\mathrm{attn}}^{2}(R,Q_{\mathrm{CLS}}^{\prime }).$$

Since the inputs and outputs share the same dimensionality, the BiQuery module can be stacked, with the number of layers *M* treated as a tunable hyper-parameter. Finally, $Q_{\mathrm{CLS}}^{{\;}^{\prime }}$ is projected by a set of category-specific linear heads $G_{c}(\cdot )$ to predict the occurrence probability of each category ${\hat{p}}_{c}$:


(10) $${\hat{p}}_{c}=\sigma (G_{c}(Q_{c}^{\prime })),\;c\in \{1,2,\ldots ,C\}.$$

In the end, the probabilities ${\hat{p}}_{c}$ and the coarse branch representation vector V are combined to form the final tile representation, which is subsequently aggregated by downstream WSI classifiers.

## 3 Experiments

**Datasets.** Experiments were conducted on the public *CDetector* dataset (Liang *et al.* 2021) and the privately collected Large-scale Cervical Screening (*L-CerScan*) dataset. Both datasets follow the Bethesda system (Pangarkar 2022), including NILM, AGC, ASC-US, LSIL, ASC-H, and HSIL. NILM is treated as the negative class, whereas the other five categories are treated as positive classes. Thus, negative and positive define the coarse classes, while the five positive subtypes define the fine-grained categories. This label scheme is used consistently at both tile and WSI levels. Dataset preprocessing and statistics are summarized in Fig. 4. Since *CDetector* does not provide WSIs, its original images are treated as RoIs and cropped into tiles using a window size (WS) of 400 px. In contrast, *L-CerScan* provides WSIs with annotated RoIs, from which tiles are cropped using WS = 800 px. To ensure lesion presence in cropped tiles, a high-performance promptable segmentor (i.e. SAM) is used to verify lesion-containing bounding boxes after cropping. Detailed preprocessing procedures and dataset information are provided in Section A.1, available as supplementary data at *Bioinformatics* online.

**Figure 4:**
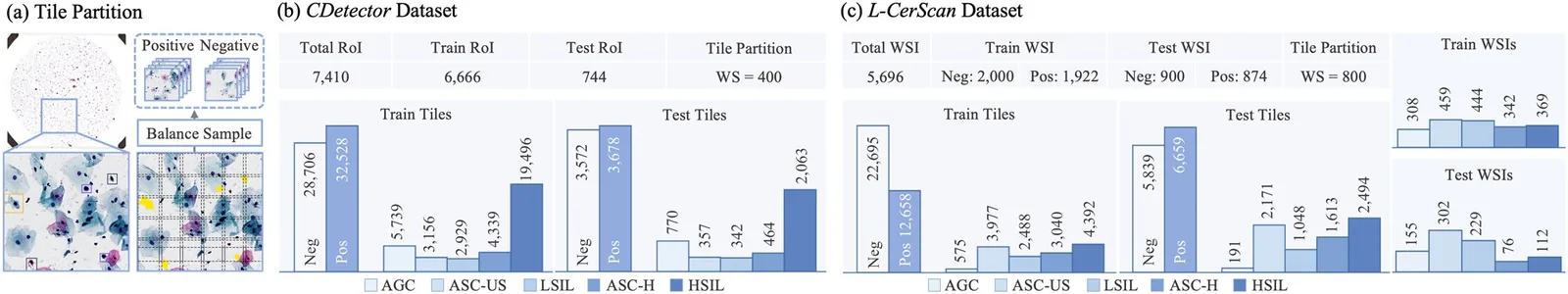
(a) Tile partitioning from cervical cytology RoIs/WSIs. Annotated lesions are processed by a segmentor and cropped at a fixed window size (WS). FMCerNet is trained with only tile-level category labels. (b) RoI and tile distributions of *CDetector*, with tiles cropped from RoIs using WS = 400 px. (c) WSI and tile distributions of *L-CerScan*, with tiles cropped from WSIs using WS = 800 px. See Section A.1, available as supplementary data at *Bioinformatics* online for more preprocessing details.

**Implementation and metrics.** In the ViT branch of LFreq-ViT, ViT-L is initialized with weights pretrained by the cervical cytopathology foundation model SmartCCS (Jiang *et al.* 2025). All comparison methods are trained with the same strategy: a learning rate of $5\times 10^{-5}$, weight decay of $1\times 10^{-3}$, and for 15 epochs. For evaluation, AUC, accuracy (Acc.), sensitivity (Sens.), and specificity (Spec.) are used for coarse classification (negative versus positive). Precision, Recall and F1-score are used to evaluate fine-grained classification: multi-label at the tile level and multi-class at the WSI level. More details are provided in Section A.2, available as supplementary data at *Bioinformatics* online.

### 3.1 Tile-level multi-label classification

Before comparing MLC heads, preliminary experiments determined the foundation model and adaptation strategy for the ViT branch of LFreq-ViT. Common vision backbones, histopathology/cytopathology foundation models were evaluated with freezing, parameter-efficient tuning, and full fine-tuning. A frozen ViT-L initialized with SmartCCS (Jiang *et al.* 2025) achieved the best performance and was therefore used as the ViT branch in all tile-level experiments. Detailed results are provided in Section B.1, available as supplementary data at *Bioinformatics* online.

The first two blocks of Table 1 show the tile-level results on *CDetector* and *L-CerScan*. LinearProbe classifies the CLS token extracted by the frozen SmartCCS using a set of linear layers, serving as the simplest baseline. Compared with LinearProbe, existing multi-label heads generally improve fine-grained recognition. Binary CHIEF (Wang *et al.* 2024b) performs competitively in coarse classification but lacks subtype prediction. Query2Label (Liu *et al.* 2021) and ML-Decoder (Ridnik *et al.* 2023) provide strong baselines, whereas MLC-NC (Tao *et al.* 2025) improves some coarse metrics but shows weaker fine-grained performance. FMCerNet achieves the best overall results on both datasets. On *CDetector*, FMCerNet achieves the best results across all metrics, with an F1-score of 65.1%, 2.6 points higher than the best competitor. On *L-CerScan*, it improves recall by 9.4 points, and rare AGC subtype F1-score by 12.7 points. These results suggest that FMCerNet more effectively captures sparse and heterogeneous lesion semantics, which is crucial for downstream WSI classification where tiles are ranked by positive probability and only the top-*k* candidates are selected for MIL aggregation. Additional tile-level analyses, including MLC label co-occurrence, error distributions, and the DTCWT frequency module, are provided in Section B.2, available as supplementary data at *Bioinformatics* online.

**Table 1:** Comparison on *CDetector* and *L-CerScan*. SmartCCS (Jiang *et al.* 2025) is used as the backbone for tile-level tasks, and RRT (Tang *et al.* 2024) as the MIL method for WSI-level tasks.^a^

	Methods	Year	Type	Coarse classification				Fine-grained classification							
				AUC	Acc	Sensitivity	Specificity	Precision	Recall	F1-score	AGC	ASC-US	LSIL	ASC-H	HSIL
*CDetector* (400 px)	LinearProbe	–	MLC	85.0	75.7	89.4	61.6	41.6	63.9	50.4	57.2	46.1	45.0	30.5	73.2
	CHIEF	2024	Binary	93.2	85.1	91.6	78.4	–							
	Query2Label	2021	MLC	92.4	83.3	93.4	72.8	50.2	82.5	61.1	74.5	54.5	56.2	38.5	82.0
	ML-Decoder	2023	MLC	92.4	84.9	94.1	75.5	52.1	79.0	62.5	73.5	55.5	63.9	38.6	80.9
	MLC-NC	2025	MLC	93.8	85.8	91.9	79.6	38.3	82.7	47.7	53.2	38.0	40.1	28.1	79.3
	Ours	–	MLC	**94.8** + 1.0	**87.2** + 1.4	**95.4** + 1.3	**82.6** + 3.0	**53.6** + 1.5	**84.4** + 1.7	**65.1** + 2.6	**75.4** + 0.9	**60.1** + 4.6	**64.2** + 0.3	**43.2** + 4.6	**82.9** + 0.9
*L-CerScan* (800 px)	LinearProbe	–	MLC	68.4	62.1	57.4	67.4	30.0	31.2	30.4	34.2	28.0	18.3	21.1	50.5
	CHIEF	2024	Binary	86.9	78.2	75.7	81.1	–							
	Query2Label	2021	MLC	82.8	75.3	75.5	75.1	39.0	55.3	44.3	42.7	46.6	41.8	36.4	54.1
	ML-Decoder	2023	MLC	84.3	75.8	76.4	75.2	41.0	54.3	46.1	41.6	49.5	47.4	34.2	58.7
	MLC-NC	2025	MLC	81.4	70.2	61.3	80.4	28.7	51.0	34.4	16.0	38.6	39.5	26.1	51.5
	Ours	–	MLC	**88.3** + 1.4	**80.9** + 2.7	**78.7** + 2.3	**83.4** + 2.3	**42.9** + 1.9	**64.7** + 9.4	**50.8** + 4.7	**55.4** + 12.7	**49.7** + 0.2	**48.0** + 0.6	**41.5** + 5.1	**59.4** + 0.7
*L-CerScan* (WSI)	SmartCCS	2025	Unsup.	89.8	89.6	84.2	**94.8**	51.7	50.7	49.9	42.0	34.8	65.4	20.9	46.2
	CHIEF	2024	Binary	90.1	90.1	89.9	90.3	53.2	51.5	51.6	51.1	45.0	57.4	26.3	40.4
	ML-Decoder	2023	MLC	90.7	89.2	85.4	93.0	54.5	53.9	53.7	59.3	39.6	60.6	23.5	49.6
	MLC-NC	2025	MLC	88.9	87.6	82.9	92.2	52.0	50.9	50.2	42.2	36.5	65.1	20.8	46.4
	Ours	–	MLC	**92.0** + 1.3	**91.2** + 1.1	**93.0** + 3.1	89.4	**60.2** + 5.7	**59.2** + 5.3	**58.6** + 4.9	**63.0** + 3.7	**49.4** + 4.4	**67.0** + 1.6	**28.0** + 1.7	**53.0** + 3.4

^a^Bold and underline denote the best and second-best results within each dataset.

### 3.2 WSI-level multi-class classification

Table 1 reports WSI-level multi-class (MC) classification results on *L-CerScan*, where all tile-level extractors are evaluated with the same RRT (Tang *et al.* 2024) MIL aggregator. The “Type” column denotes the training paradigm used to obtain each tile representation, such as unsupervised pretraining for SmartCCS and binary supervision for CHIEF. The construction of tile representations for the baseline extractors is detailed in Section B.3, available as supplementary data at *Bioinformatics* online.

As shown in the last block of Table 1, directly using SmartCCS features yields high specificity but low sensitivity, causing clinically unacceptable misses of positive WSIs. Its limited fine-grained recognition further suggests that unsupervised tile representations are insufficient for WSI-level MC classification. CHIEF improves coarse classification but remains constrained by its binary design. Among multi-label tile extractors, ML-Decoder provides the strongest baseline, achieving an F1-score of 53.7%. FMCerNet achieves the best overall WSI-level performance, with clear improvements over its binary counterpart CHIEF in key metrics such as sensitivity and F1-score, especially for subtype classification of AGC, LSIL, and HSIL. These results indicate that the fine-grained tile representations learned by FMCerNet better support MIL-based WSI diagnosis.

### 3.3 Ablation, efficiency, and interpretability

Figure 5 summarizes complementary evaluations of FMCerNet: (1) tile-level efficiency, module ablation, and hyperparameter sensitivity; (2) WSI-level aggregation, crop size, and feature extractor analysis; (3) salient map M interpretability. At the tile level, larger inputs in existing MLC heads are directly fed into ViT-L, incurring substantial overhead for modest gains. FMCerNet instead processes 1024 px inputs at 64.2 tiles/s and achieves the best performance (Fig. 5a). Component ablations show complementary roles of the MLC branch, DTCWT block, and salient map reweighting (MapRe.), with any module removal weakening coarse classification (Fig. 5b). Moreover, removing DTCWT or MapRe. decreases MLC precision but increases recall, suggesting that both modules improve positive tile detection while making subtype prediction more selective, yielding more robust precision and F1-score at the cost of recall. Hyperparameter analysis identifies K=3 and M=2 as the best setting for sensitivity, with robust performance across different stack numbers (Fig. 5c).

**Figure 5:**
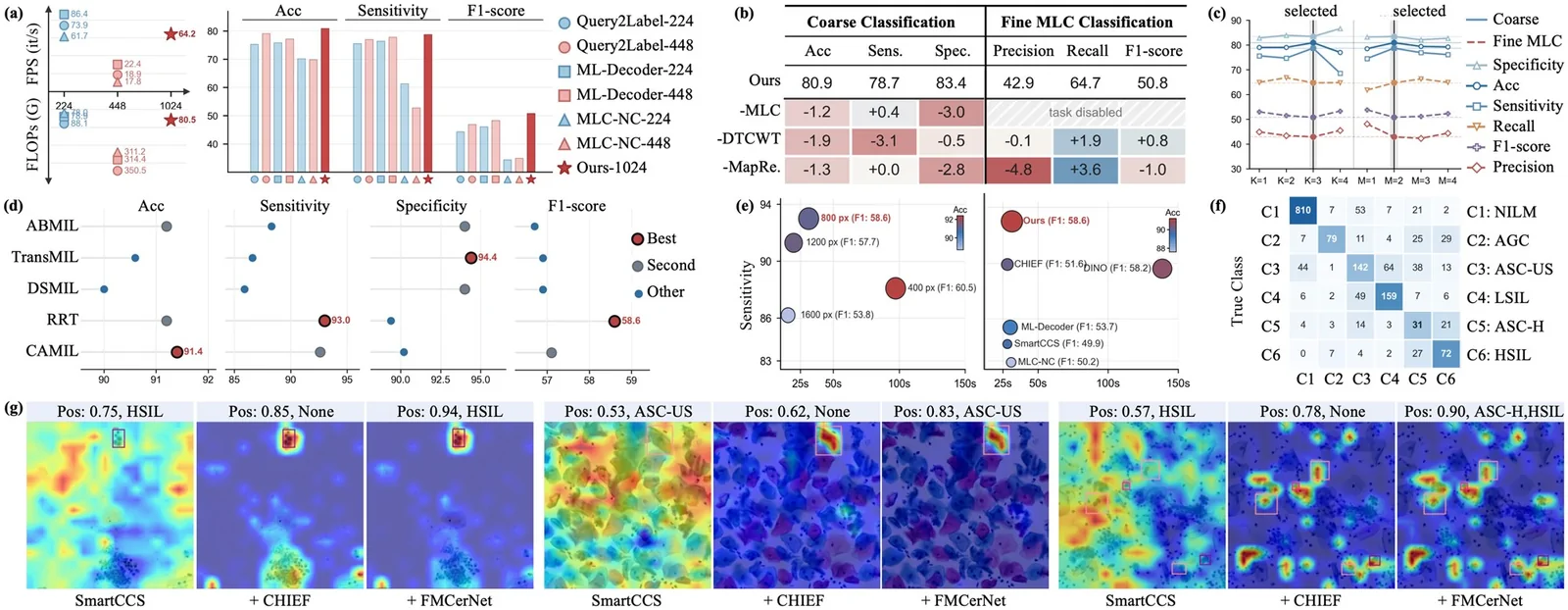
Tile-level ablation studies: (a) Comparison under WS = 800 px with varying input sizes, where FMCerNet uses a 1024 px input and achieves the best trade-off between inference cost and performance. (b) Performance degradation from ablating the proposed modules. (c) Sensitivity analysis of stack numbers *K* and *M* in the Frequency Refine Block and BiQuery Module. WSI-level ablation studies: (d) Comparison of MIL methods using FMCerNet as the tile-level extractor. (e) Effects of crop WS under a fixed 1024 px input and tile-level extractor choice on WSI classification. (f) Six-class confusion matrix under the best WSI-level setting. Salient map visualization: (g) Sparse cells with sparse lesions, dense cells with sparse lesions, and dense cells with dense multi-type lesions.

At the WSI level, Fig. 5d–f provides an integrated assessment of FMCerNet’s robustness and practical utility for WSI classification. Figure 5d compares five representative MIL aggregators, including ABMIL (Ilse *et al.* 2018), TransMIL (Shao *et al.* 2021), DSMIL (Li *et al.* 2021), RRT (Tang *et al.* 2024), and CAMIL (Mao *et al.* 2025). RRT provides the strongest sensitivity and F1-score, supporting its use for WSI-level diagnosis. Figure 5e compares the effects of crop size and tile-level extractor on WSI-level MC classification performance and inference speed on the same slide. With a fixed 1024 px tile input, larger WS speeds up WSI inference but reduces sensitivity, making WS = 800 px a strong trade-off between sensitivity and speed. Other classification-trained tile extractors show similar speed but much lower performance. For detection-based comparison, DINO (Zhang *et al.* 2023) trained on *L-CerScan* cell annotations retrieves the top-*k* suspicious positive cells per WSI, which are cropped and encoded by SmartCCS before MIL aggregation. It matches FMCerNet in performance but is substantially slower, highlighting FMCerNet’s efficiency advantage for WSI classification. Figure 5f presents the six-class confusion matrix of FMCerNet from the last block of Table 1, where most errors occur between visually similar abnormal categories, such as ASC-US/LSIL and ASC-H/HSIL, reflecting common challenges in cervical cytology screening.

Finally, Fig. 5g evaluates the interpretability of the salient map M in three representative scenarios. The comparison includes frozen SmartCCS, SmartCCS with the CHIEF binary head, and the full FMCerNet. For SmartCCS, M is computed by the cosine similarity between the CLS token and tile representations. As shown, vanilla SmartCCS shows diffuse attention, and CHIEF suppresses background noise but remains uncertain in dense-background and multi-type lesions. FMCerNet further increases positive confidence, suggesting that fine-grained MLC supervision improves tile representations and reinforces the binary branch.

## 4 Conclusion

In this paper, we propose the Frequency-Aware Multi-Label Learning Network (FMCerNet) for fine-grained cervical cytology screening. FMCerNet serves as a tile-level feature extractor with a Large-Field Frequency-Enhanced ViT (LFreq-ViT) backbone for efficient large-field input processing and a Coarse-to-Fine (C2F) head that uses the salient map from a coarse binary branch to guide fine-grained multi-label classification. By incorporating the Dual-Tree Complex Wavelet Transform (DTCWT) in the LFreq-ViT backbone, FMCerNet overcomes the input-size limitations of foundation models and enhances the extraction of semantically rich and discriminative features. By leveraging the salient map generated by CHIEF, C2F provides hierarchical supervision that enables FMCerNet to achieve strong coarse and fine-grained classification performance. With these components, FMCerNet achieves high accuracy in both tile- and WSI-level classification, assisting pathologists in efficiently screening positive cases. Extensive experiments on the public *CDetector* dataset and our private *L-CerScan* dataset demonstrate that FMCerNet outperforms existing methods in coarse binary screening and fine-grained multi-class diagnosis while maintaining high inference speed. Salient map visualizations further show improved attribution interpretability for lesion identification, supporting FMCerNet as a scalable, accurate, and interpretable solution for automated cervical cytology screening.

## Supplementary Material

btag759_Supplementary_Data

## Data Availability

The public CDetector dataset used in this study is available at https://github.com/CVIU-CSU/ComparisonDetector. The L-CerScan dataset is not publicly available due to patient privacy concerns and restrictions imposed by ethical approval.
