# Supplementary material for: FMCerNet: frequency-aware multi-label learning for fine-grained cervical cytology screening
Source: Bioinformatics. 2026 Oct 19;42(10):btag759. doi: 10.1093/bioinformatics/btag759 (PMC13716490; doi:10.1093/bioinformatics/btag759)
Supplement: Supplementary file 1 [file btag759_supplementary_data.pdf]

FMCerNet: Frequency-Aware Multi-Label Learning for Fine-Grained Cervical Cytology Screening

Supplementary Material

A. Supplementary Experimental Settings

A.1. Dataset Information and Preprocessing

The publicly available dataset *CDetector* was collected for abnormal cervical lesion detection annotated by expert pathologists. After removing error-annotated bounding boxes (bboxes) with width or height no larger than 5 pixels, the dataset contains 50,710 valid bboxes over 6,666 training and 744 test images of varying sizes. The original bboxes cover 11 categories, including lesion classes and negative NILM classes. To focus on positive screening and lesion subtype classification, these categories are remapped according to the Bethesda system (Nayar and Wilbur, 2015), as shown in Tab. S1. The *CDetector* preprocessing pipeline is shown in Fig. S1(a). For each image with varying width and height, annotated bbox are sequentially used as prompts for SAM (Kirillov et al., 2023) segmentation. The image is then cropped by a sliding window with a fixed size of 400×400 px and a stride of 50 px. Tiles containing lesions are labeled as positive, and the remaining tiles are labeled as negative. The multi-label of each positive tile is obtained by removing duplicate category labels from the bbox contained in that tile. Notably, this dataset does not provide WSI-level sources.

Thus, 5,696 cervical cytology WSIs were collected to form *L-CerScan*, including 2,900 negative and 2,796 positive slides, with positive slides further annotated by four experienced pathologists. Each patient contributed one WSI. The training and test sets were partitioned at the patient level with no patient overlap, and all tiles extracted from each WSI were assigned to the same split. Fig. S1(b) shows the WSI-level preprocessing pipeline. For each WSI, pathologists delineate variable-size RoIs and mark all lesions within them using bboxes, leaving regions without bbox annotations as normal. Since RoIs range from 4,000 px to over 10,000 px, SAM cannot process them directly. Bboxes within each RoI are sorted by area and organized into a BBox Stack, from which 1024×1024 px regions centered on individual bboxes are sequentially cropped and fed into SAM to obtain lesion masks. RoI tiles are then cropped using an 800 px window with a 50 px stride. A tile with k masks is labeled positive if at least one mask area exceeds $\theta = 50$, and negative otherwise, following the same rule as *CDetector*. Notably, bbox are sorted by area in descending order to prevent masks of small bboxes from being overwritten by masks of larger enclosing bboxes.

Under the balanced sampling strategy, all positive tiles are retained, while negative tiles from each RoI are randomly shuffled and capped at 15. To further enrich the negative samples, additional tiles are randomly cropped from negative slides and filtered, retaining at most 10 and 15 tiles per slide for the training and test sets, respectively.

It should be noted that bounding box annotations in *L-CerScan* enable comparison of classification- and detection-based region feature extraction for WSI classification and are used to derive tile labels. However, FMCerNet can be trained directly on category-labeled tiles without instance-level bounding boxes.

Table S1 *CDetector* category mapping and instance annotation counts.

Original class	Mapped class	Train instances	Test instances
ascus	ASC-US	1755	212
lsil	LSIL	1399	165
asch	ASC-H	3858	402
hsil	HSIL	26028	2793
scc	HSIL	1963	228
agc	AGC	4900	653
trichomonas	NILM	4937	477
candida	NILM	324	26
flora	NILM	123	24
herps	NILM	262	35
actinomyces	NILM	130	16
Total	–	45679	5031

A.2. Implementation Details and Evaluation Protocol

Regarding hyperparameter settings, the number of stacked DTCWT blocks (K) and BiQuery layers (M) are set to 3 and 2, respectively. For training details, all experiments are conducted on 8 NVIDIA A800 GPUs with a per-GPU batch size of 128 using the AdamW optimizer and a learning rate scheduler combining LinearLR and CosineAnnealingLR. The ViT branch remains fully frozen during training. More detailed configurations are available in our released code, which provides instructions for reproducing all experiments.

For performance evaluation at the tile level, coarse classification uses the sigmoid output of the binary branch as the positive score for methods with a dedicated binary branch, and the maximum predicted probability across the five abnormal categories for methods without one. Precision, recall, and F1-score for fine-grained multi-label classification are macro-averaged over these five categories. At the WSI level, coarse classification is derived from the predicted class, selected by argmax over the six class probabilities, with NILM treated as negative and all abnormal categories as positive. Fine-grained multi-class classification comprises six classes, including NILM, over which precision, recall, and F1-score are macro-averaged.

A.3. Training Loss Settings

For tile-level training, the C2F head jointly optimizes binary screening and fine-grained multi-label classification. Let B denote the batch size, y_i the binary label, and y_{ic} the label for abnormal category c, with $C = 5$. The corresponding predicted probabilities are $\hat{p}_i$ and $\hat{p}_{ic}$. The binary branch uses binary cross-entropy:

$$\mathcal{L}_{\text{binary}} = -\frac{1}{B}\sum_{i=1}^{B}\left[y_i \log \hat{p}_i + (1-y_i)\log(1-\hat{p}_i)\right]. \qquad \text{(S1)}$$

Table S2 Binary classification comparison of CHIEF on *L-CerScan* (800 px). ResNet50 and ConvNeXt-B are fully fine-tuned, other common and histopathology backbones are adapted with LoRA (Hu et al., 2022), and cytopathology backbones are frozen with only the CHIEF binary head trained. LFreq-ViT builds on frozen SmartCCS and incorporates DTCWT for large input image processing. Bold and underline indicate the best and second-best results, respectively.

Type	Backbone	Params (M) Total/Trainable	Input Size	FLOPs(G)	FPS(it/s)	AUC	Accuracy	Sensitivity	Specificity
Common	ResNet50 (He et al., 2016)	26.66/26.66	224	4.29	227.93	76.46	66.35	52.83	81.76
	ConvNeXt-B (Liu et al., 2022)	88.49/88.49	224	15.40	163.78	86.38	75.01	65.49	**85.87**
	ViT-L (Dosovitskiy et al., 2021)	305.79/2.10	224	20.44	55.10	75.71	68.11	70.97	64.86
	DINOv2 (Oquab et al., 2023)	306.47/2.10	224	26.61	48.44	84.88	75.91	75.43	76.45
	DINOv3 (Siméoni et al., 2025)	307.59/4.46	224	61.79	45.96	80.22	69.98	59.08	82.41
Histopathology	CTransPath (Wang et al., 2022)	28.18/28.18	224	4.41	159.53	83.39	74.00	69.65	78.95
	UNI2-H (Chen et al., 2024)	687.18/5.79	224	181.90	31.50	86.67	77.33	73.04	82.22
	Virchow2 (Zimmermann et al., 2024)	637.42/6.18	224	166.18	29.78	87.14	78.45	77.55	79.48
	GPFM (Ma et al., 2026)	307.31/4.08	224	78.86	52.91	83.14	73.64	68.24	79.81
	GenBio-PathFM (Kapse et al., 2026)	1144.68/10.99	224	688.97	8.95	86.76	77.32	73.58	81.59
Cytopathology	SmartCCS (Jiang et al., 2025)	304.15/0.92	224	78.05	76.82	88.08	79.19	76.38	82.39
	CytoFM (Ivezic et al., 2025)	86.46/0.66	224	16.99	177.13	77.65	66.31	53.31	81.13
	UniCAs (Jiang et al., 2026)	304.70/0.92	224	20.20	67.18	81.55	71.75	64.00	80.58
	LFreq-ViT (Ours)	307.25/4.02	1024	80.02	65.14	**88.37**	**79.72**	**79.13**	80.39

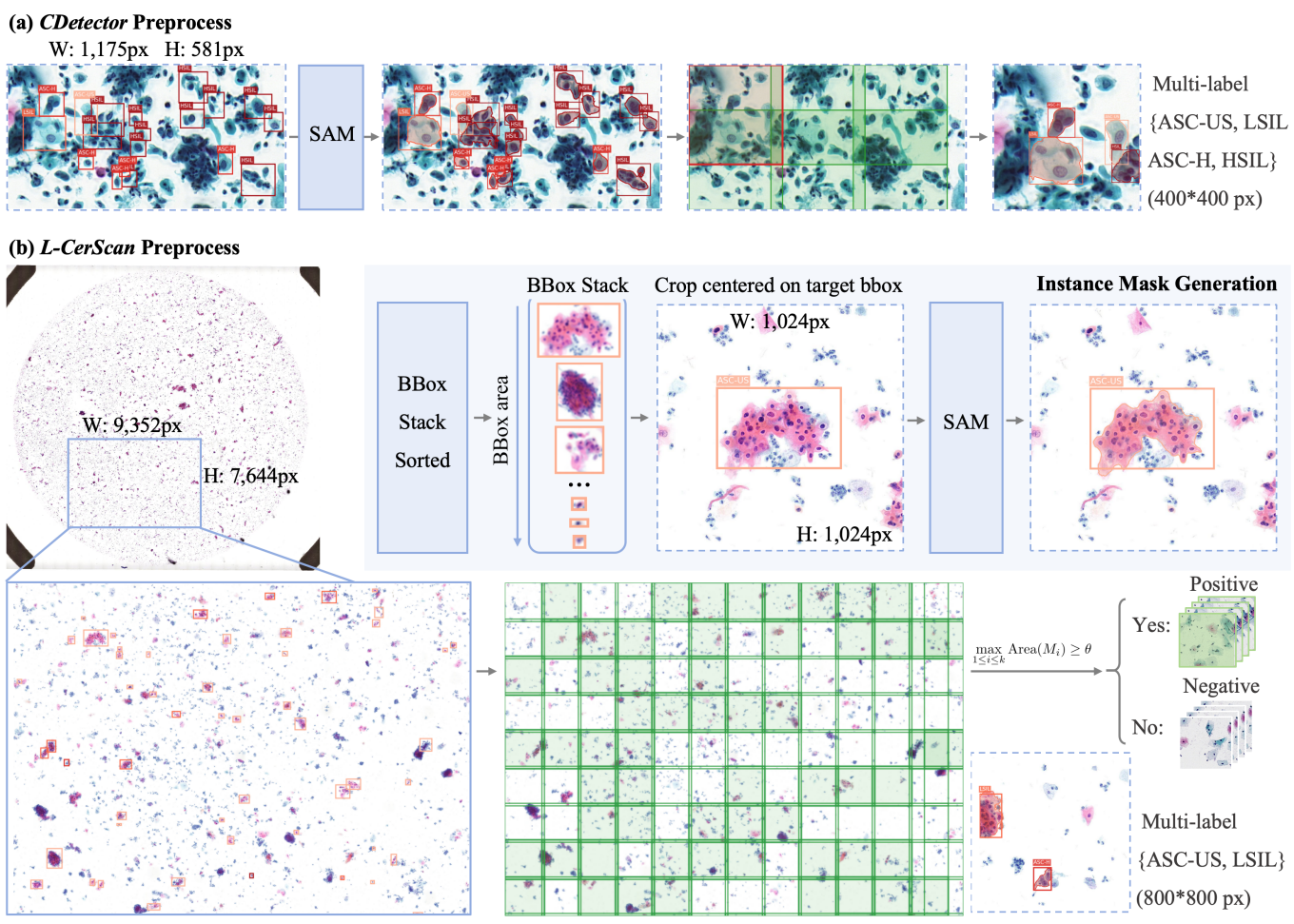

Figure S1 Preprocessing pipeline of *CDetector* (**(a)**) and *L-CerScan* (**(b)**).

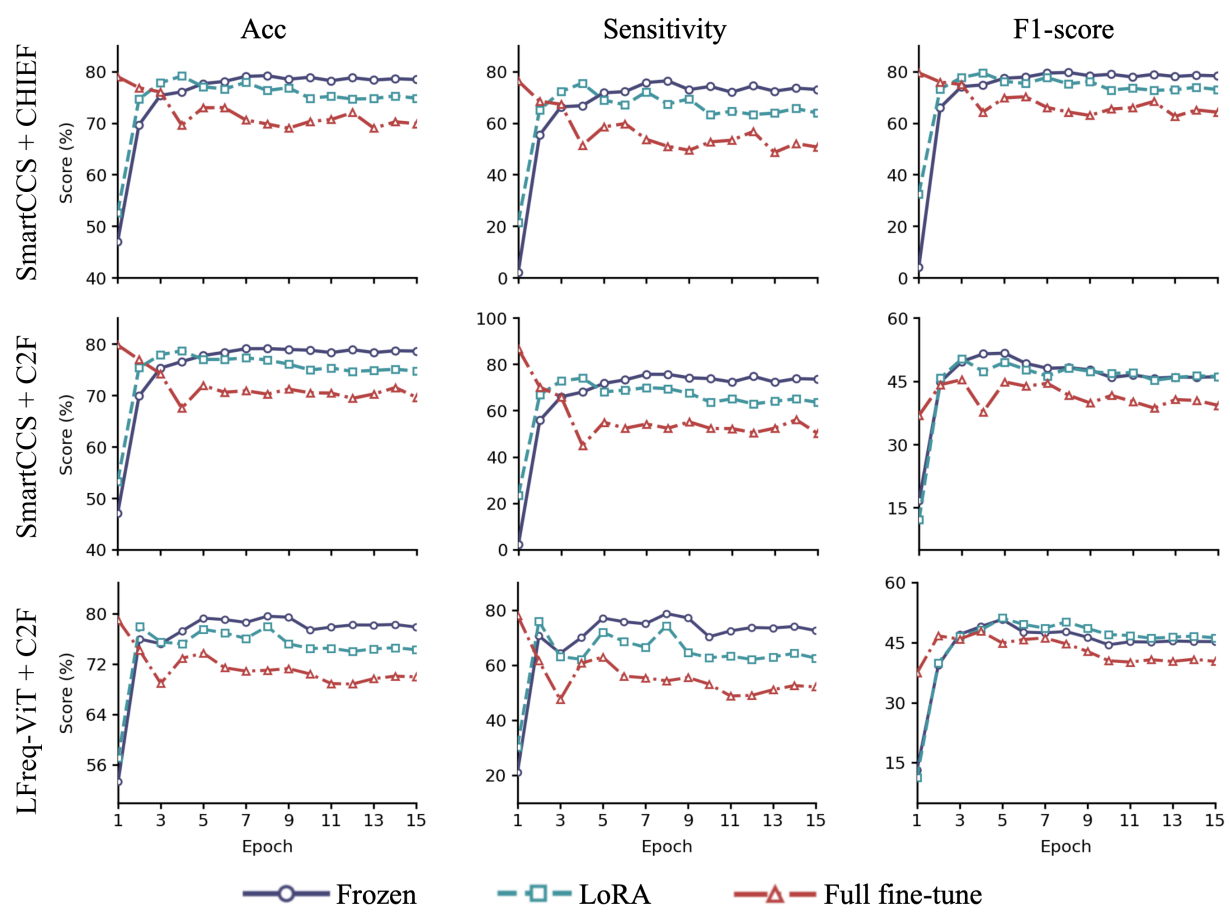

Figure S2 Performance of different adaptation strategies for the ViT branch.

The multi-label branch uses asymmetric loss with focusing parameters $\gamma_{\text{pos}} = 0$ and $\gamma_{\text{neg}} = 2$, without probability clipping:

$$\mathcal{L}_{\text{multi}} = -\frac{1}{BC}\sum_{i=1}^{B}\sum_{c=1}^{C}\Big[y_{ic}\log \hat{p}_{ic} + (1-y_{ic})\hat{p}_{ic}^{2}\log(1-\hat{p}_{ic})\Big]. \tag{S2}$$

The asymmetric focusing weights are treated as constants during backpropagation, and logarithm arguments are lower-bounded by 10^{-5} for numerical stability. Both losses are evaluated over all tiles, with negative tiles assigned all-zero multi-label targets. The overall training objective is

$$\mathcal{L} = \lambda_{\text{binary}}\mathcal{L}_{\text{binary}} + \lambda_{\text{multi}}\mathcal{L}_{\text{multi}}, \quad \lambda_{\text{binary}} = \lambda_{\text{multi}} = 1. \tag{S3}$$

B. Supplementary Experimental Results

B.1. ViT Branch Initialization and Adaptation

To determine the ViT branch initialization, common vision backbones were compared with histopathology and cytopathology foundation models using the CHIEF binary classification head, as positive slide screening is the primary objective. As shown in Table S2, SmartCCS achieved the highest AUC (88.08) and accuracy (79.19) among standalone backbones, while maintaining comparable sensitivity to Virchow2 (76.38 vs. 77.55) with less than half the computational cost (78.05 vs. 166.18 GFLOPs). Although individual models achieved higher sensitivity or specificity, none matched SmartCCS in overall discrimination and accuracy. With the ViT branch initialized by SmartCCS pretrained weights, incorporating the frequency branch into LFreq-ViT further improves AUC, accuracy, and sensitivity to 88.37, 79.72, and 79.13, respectively. Notably, ResNet50 and ConvNeXt-B were fully fine-tuned due to their smaller parameter sizes and convolutional architectures. Other transformer-based backbones were adapted with LoRA to reduce overfitting and training overhead, except for cytopathology specific backbones that

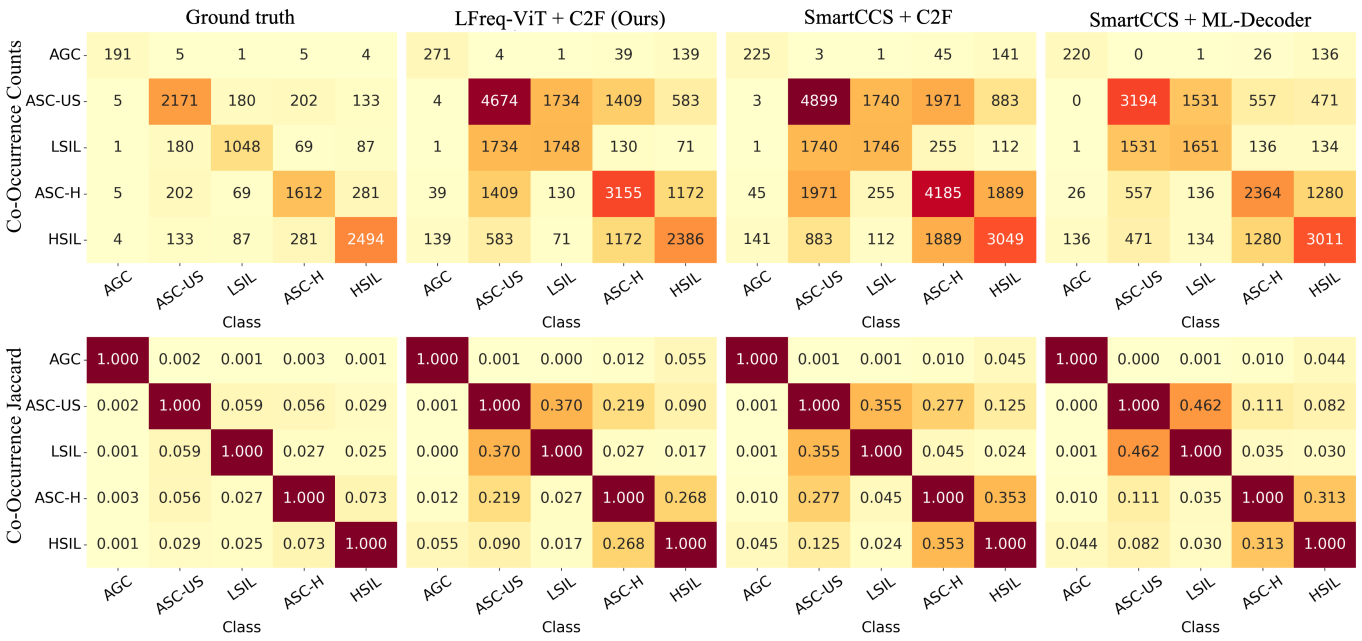

Figure S3 Ground-truth and predicted pairwise co-occurrence counts (top) and Jaccard coefficients (bottom) for the five positive categories.

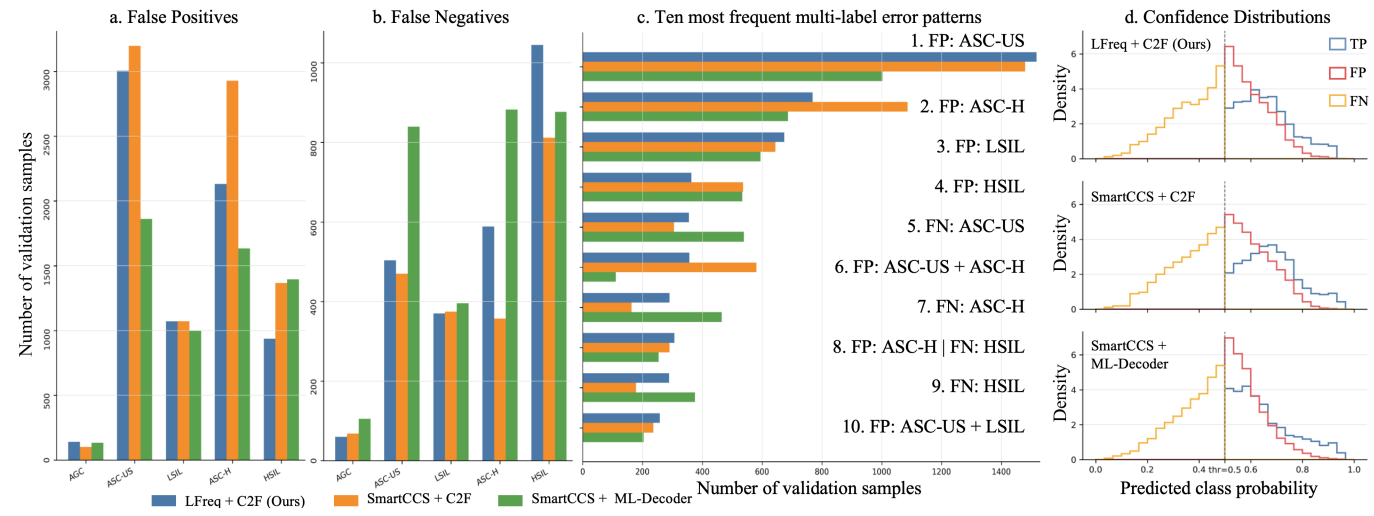

Figure S4 Error analysis across three model configurations includes (a) class-wise false positives, (b) class-wise false negatives, (c) frequent multi-label error patterns, and (d) confidence distributions.

Table S3 Comparison of frequency-domain operators on the test set. The best result for each metric is highlighted in bold.

Frequency operator	Acc	Sensitivity	Specificity	Precision	Recall	F1-score	FPS (it/s) ↑
DWT-Haar	77.3	69.7	**85.9**	**43.2**	64.8	**51.8**	75.2
FFT	79.2	75.9	82.9	40.8	**69.3**	51.1	**75.9**
DTCWT	**79.3**	**77.1**	81.9	42.9	64.7	50.8	64.2

were kept frozen. Based on the SmartCCS initialized ViT branch, we further evaluated frozen, LoRA-based, and full fine-tuning strategies under three model configurations: the CHIEF binary head, the proposed C2F head, and the full LFreq-ViT backbone. As shown in Fig. S2, freezing the ViT backbone consistently achieved the best and most stable accuracy, sensitivity, and F1-score across all configurations. Full fine-tuning exhibited stronger overfitting, while LoRA incurred additional training costs but provided no improvement over freezing.

B.2. Additional Tile-Level Analyses

B.2.1. Multi-Label Co-Occurrence Analysis

To assess whether the models captured dependencies among the five positive categories, we compared predicted pairwise co-occurrence patterns with ground truth (GT) on the test set. As shown in Fig. S3, the upper row presents raw co-occurrence counts. Diagonal entries denote the number of samples containing each category, whereas off-diagonal entries denote the number containing both categories. The lower row presents Jaccard coefficients, defined as the number of samples containing both categories divided by the number containing either category. The GT exhibited relatively sparse pairwise co-occurrence, with ASC-H–HSIL showing the highest off-diagonal Jaccard coefficient. By contrast, all three methods produced denser co-occurrence patterns, particularly for ASC-US–LSIL and ASC-H–HSIL, which may be attributed to the morphological continuity among adjacent lesion subtypes that causes models to simultaneously activate neighboring categories. Pearson correlation and Jaccard mean absolute error (MAE) were calculated over the ten unique category pairs. Our LFreq-ViT backbone with the C2F classification head achieved a Pearson correlation of 0.8852 and the lowest Jaccard MAE of 0.0802. SmartCCS with C2F achieved the highest correlation of 0.9435 but a larger MAE of 0.0964. This result indicates that it preserved the relative co-occurrence structure while overestimating association strength. SmartCCS with ML-Decoder achieved a correlation of 0.8102 and a Jaccard MAE of 0.0815. Overall, FMCerNet achieved the smallest discrepancy in co-occurrence estimation while maintaining strong agreement with the GT pattern.

B.2.2. Error Distribution Analysis

We further examined the fine-grained label prediction errors of the three methods on the test set (Fig. S4). As shown in Fig. S4(a,b), false positives (FPs) were mainly associated with ASC-US and ASC-H, whereas AGC showed fewer error instances due to its lower prevalence. LFreq-ViT+C2F produced 7,285 label-level FPs and 2,567 false negatives (FNs), compared with 8,669 FPs and 2,081 FNs for SmartCCS+C2F and 6,025 FPs and 3,101 FNs for SmartCCS+ML-Decoder. These results reveal a precision-recall trade-off: SmartCCS+C2F identified more positive labels but introduced more false positives, whereas SmartCCS+ML-Decoder reduced FPs at the cost of more missed positives. For LFreq-ViT+C2F, FNs were mainly concentrated in HSIL, while FPs were dominated by ASC-US and ASC-H.

The structured error patterns in Fig. S4(c) further show that isolated ASC-US overprediction was the most frequent error across all three methods, followed by false-positive ASC-H and LSIL predictions. Composite errors, including simultaneous ASC-US and ASC-H overprediction and ASC-H–HSIL confusion, indicate that errors were correlated across labels and concentrated among morphologically related categories. The confidence distributions in Fig. S4(d) further highlights the more balanced error profile of LFreq-ViT+C2F. Compared with SmartCCS+C2F, our method reduces label-level false positives from 8,669 to 7,285, while reducing false negatives from 3,101 to 2,567 compared with SmartCCS+ML-Decoder. Therefore, LFreq-ViT+C2F achieves a better balance between suppressing false positives and retaining positive findings. Across all three methods, FP and FN confidence scores are concentrated near the decision boundary, with 49.2–60.8% of FPs and 42.4–46.3% of FNs falling within $[0.4, 0.6]$. The accumulation of errors around the threshold suggests that many misclassifications correspond to borderline cases rather than unequivocally incorrect predictions. Overall, these analyses indicate that improving discrimination of morphologically overlapping lesions could further enhance multi-label recognition.

B.2.3. Ablation of Frequency Module

To evaluate different frequency decomposition strategies, DTCWT is compared with a two-level Haar discrete wavelet transform based on multiresolution decomposition (Mallat, 1989) and an FFT-based spectral operator (Cooley and Tukey, 1965), where the centered magnitude spectrum is partitioned into three radial frequency bands. As shown in Tab. S3, DTCWT achieves the best accuracy and sensitivity, which are particularly important for cervical cytology screening. Compared with FFT, DTCWT provides

higher sensitivity while maintaining comparable accuracy, whereas compared with DWT-Haar, it better captures subtle lesion-related features by improving sensitivity. Although DTCWT introduces moderate computational overhead, it maintains competitive inference speed and achieves the best balance between performance and efficiency, motivating its adoption in LFreq-ViT.

B.3. Tile Representation Construction for WSI Baselines

To construct the WSI representation, a method-specific tile feature extraction strategy was adopted according to the output structure of each tile-level extractor. For SmartCCS, the `[CLS]` token of each selected tile was directly used as its feature vector. For CHIEF, the binary branch feature was retained, and tiles were ranked by their predicted positive probabilities. Since ML-Decoder and MLC-NC lack a binary branch, each tile representation was obtained by summing the three positive category features with the highest predicted probabilities, and tiles were ranked according to their maximum positive category probability. For FMCerNet, the binary branch feature was concatenated with the predicted probability vector of the five positive categories to preserve both general abnormality information and category-specific diagnostic evidence. Tile vectors were ranked by binary probabilities, and the top-K vectors ($K = 1000$ by default) were stacked into a fixed-length WSI representation, with zero-padding applied when necessary.

References

R. J. Chen, T. Ding, M. Y. Lu, D. F. Williamson, G. Jaume, B. Chen, A. Zhang, D. Shao, A. H. Song, M. Shaban, et al. Towards a general-purpose foundation model for computational pathology. *Nature Medicine*, 2024.

J. W. Cooley and J. W. Tukey. An algorithm for the machine calculation of complex fourier series. *Mathematics of computation*, 19(90):297–301, 1965.

A. Dosovitskiy, L. Beyer, A. Kolesnikov, D. Weissenborn, X. Zhai, T. Unterthiner, M. Dehghani, M. Minderer, G. Heigold, S. Gelly, J. Uszkoreit, and N. Houlsby. An image is worth 16x16 words: Transformers for image recognition at scale. In *Proc. Int. Conf. Learn. Represent.*, May 2021.

K. He, X. Zhang, S. Ren, and J. Sun. Deep residual learning for image recognition. In *Proc. IEEE/CVF Conf. Comput. Vis. Pattern Recognit. (CVPR)*, pages 770–778, Jun. 2016.

E. J. Hu, Y. Shen, P. Wallis, Z. Allen-Zhu, Y. Li, S. Wang, L. Wang, W. Chen, et al. Lora: Low-rank adaptation of large language models. *Iclr*, 1(2):3, 2022.

V. Ivezic, A. Radhachandran, E. Redekop, S. Athreya, D. Lee, V. Sant, C. Arnold, and W. Speier. Cytofm: The first cytology foundation model. In *Proceedings of the Computer Vision and Pattern Recognition Conference*, pages 4749–4757, 2025.

H. Jiang, C. Jin, H. Lin, Y. Zhou, X. Wang, J. Ma, L. Ding, J. Hou, R. Liu, Z. Chai, et al. Generalizable cervical cancer screening via large-scale pretraining and test-time adaptation. *arXiv:2502.09662*, 2025.

H. Jiang, J. Cai, Z. Shen, M. Xu, M. Fei, H. Huang, X. Wang, R. Bi, D. Shen, L. Zhang, et al. Unicas: A foundation model for cervical cytology screening. *Cell Reports Medicine*, 7(1), 2026.

S. Kapse, M. Aygün, E. Cole, E. Lundberg, L. Song, and E. P. Xing. Genbio-pathfm: A state-of-the-art foundation model for histopathology. *bioRxiv*, pages 2026–03, 2026.

A. Kirillov, E. Mintun, N. Ravi, H. Mao, C. Rolland, L. Gustafson, T. Xiao, S. Whitehead, A. C. Berg, W.-Y. Lo, et al. Segment anything. In *Proc. IEEE/CVF Int. Conf. Comput. Vis. (ICCV)*, pages 4015–4026, Oct. 2023.

Z. Liu, H. Mao, C.-Y. Wu, C. Feichtenhofer, T. Darrell, and S. Xie. A convnet for the 2020s. In *Proceedings of the IEEE/CVF conference on computer vision and pattern recognition*, pages 11976–11986, 2022.

J. Ma, Z. Guo, F. Zhou, Y. Wang, Y. Xu, J. Li, F. Yan, Y. Cai, Z. Zhu, C. Jin, et al. A generalizable pathology foundation model using a unified knowledge distillation pretraining framework. *Nature Biomedical Engineering*, 10(3):545–564, 2026.

S. G. Mallat. A theory for multiresolution signal decomposition: the wavelet representation. *IEEE transactions on pattern analysis and machine intelligence*, 11(7):674–693, 1989.

R. Nayar and D. C. Wilbur. *The Bethesda system for reporting cervical cytology: definitions, criteria, and explanatory notes.* Springer, 2015.

M. Oquab, T. Darcet, T. Moutakanni, H. V. Vo, M. Szafraniec, V. Khalidov, P. Fernandez, D. Haziza, F. Massa, A. El-Nouby, R. Howes, P.-Y. Huang, H. Xu, V. Sharma, S.-W. Li, W. Galuba, M. Rabbat, M. Assran, N. Ballas, G. Synnaeve, I. Misra, H. Jegou, J. Mairal, P. Labatut, A. Joulin, and P. Bojanowski. Dinov2: Learning robust visual features without supervision. *arXiv:2304.07193*, 2023.

O. Siméoni, H. V. Vo, M. Seitzer, F. Baldassarre, M. Oquab, C. Jose, V. Khalidov, M. Szafraniec, S. Yi, M. Ramamonjisoa, et al. Dinov3. *arXiv preprint arXiv:2508.10104*, 2025.

X. Wang, S. Yang, J. Zhang, M. Wang, J. Zhang, W. Yang, J. Huang, and X. Han. Transformer-based unsupervised contrastive learning for histopathological image classification. *Medical image analysis*, 81:102559, 2022.

E. Zimmermann, E. Vorontsov, J. Viret, A. Casson, M. Zelechowski, G. Shaikovski, N. Tenenholtz, J. Hall, D. Klimstra, R. Yousfi, et al. Virchow2: Scaling self-supervised mixed magnification models in pathology. *arXiv preprint arXiv:2408.00738*, 2024.
